# Activation of microglial cells triggers a release of brain-derived neurotrophic factor (BDNF) inducing their proliferation in an adenosine A_2A_ receptor-dependent manner: A_2A_ receptor blockade prevents BDNF release and proliferation of microglia

**DOI:** 10.1186/1742-2094-10-16

**Published:** 2013-01-30

**Authors:** Catarina Gomes, Raquel Ferreira, Jimmy George, Rui Sanches, Diana I Rodrigues, Nélio Gonçalves, Rodrigo A Cunha

**Affiliations:** 1 Center for Neuroscience and Cell Biology, University of Coimbra, Largo Marquês de Pombal, Coimbra, 3004-517, Portugal; 2 FMUC - Faculty of Medicine, University of Coimbra, Coimbra, 3004-504, Portugal; 3 Faculty of Pharmacy, University of Coimbra, Coimbra, 3000-548, Portugal

**Keywords:** Adenosine A_2A_ receptors, Neuroinflammation, Microglia, BDNF

## Abstract

**Background:**

Brain-derived neurotrophic factor (BDNF) has been shown to control microglial responses in neuropathic pain. Since adenosine A_2A_ receptors (A_2A_Rs) control neuroinflammation, as well as the production and function of BDNF, we tested to see if A_2A_R controls the microglia-dependent secretion of BDNF and the proliferation of microglial cells, a crucial event in neuroinflammation.

**Methods:**

Murine N9 microglial cells were challenged with lipopolysaccharide (LPS, 100 ng/mL) in the absence or in the presence of the A_2A_R antagonist, SCH58261 (50 nM), as well as other modulators of A_2A_R signaling. The BDNF cellular content and secretion were quantified by Western blotting and ELISA, A_2A_R density was probed by Western blotting and immunocytochemistry and cell proliferation was assessed by BrdU incorporation. Additionally, the A_2A_R modulation of LPS-driven cell proliferation was also tested in primary cultures of mouse microglia.

**Results:**

LPS induced time-dependent changes of the intra- and extracellular levels of BDNF and increased microglial proliferation. The maximal LPS-induced BDNF release was time-coincident with an LPS-induced increase of the A_2A_R density. Notably, removing endogenous extracellular adenosine or blocking A_2A_R prevented the LPS-mediated increase of both BDNF secretion and proliferation, as well as exogenous BDNF-induced proliferation.

**Conclusions:**

We conclude that A_2A_R activation plays a mandatory role controlling the release of BDNF from activated microglia, as well as the autocrine/paracrine proliferative role of BDNF.

## Background

Neurodegenerative pathologies, such as Alzheimer’s or Parkinson’s disease, progress with a sustained, chronic neuroinflammatory process [1,2]. Microglial cells, which display specialized immune functions in the central nervous system, play a crucial role in neuroinflammation [2,3], undergoing a process globally known as microgliosis. Microgliosis encompasses morphological changes, cell proliferation and modifications in the synthesis and secretion of both pro- and anti-inflammatory substances [4], whose temporal balance determines the contention or the perpetuation of neuroinflammation. Thus, neuroinflammation is a tightly regulated process involving several modulators, including adenosine [5]. The relation between adenosine and neuroinflammation is heralded by the key role of adenosine A_2A_ receptors (A_2A_R) in peripheral inflammation [6], as well as by the localization of A_2A_R in microglial cells and the ability of A_2A_R to control several microglial functions, namely chemotaxis [7] and the production of inflammatory mediators [8,9].

One of the most notable modulatory properties of adenosine is the ability of A_2A_R activation to potentiate the production, secretion and effects of different trophic factors [10]. This effect has been considered neuroprotective, in accordance with the ability of trophic factors to prevent neuronal damage or to promote neuronal repair [11-13]. However, it seems to contradict the well-described neuroprotective role mediated by A_2A_R antagonism [14,15]. BDNF (brain-derived neurotrophic factor), which is mostly recognized as a pro-survival factor, has also been shown to support microglial activation *in vivo*[16]. This could result in a potential amplification of microgliosis and neuroinflammation, as occurs in neuropathic pain [17,18], with BDNF release resulting either through neuronal activity-dependent exocytosis or from microglia using an exocytotic pathway or a constitutive release pathway present in all cell types (reviewed in [19]), both of which are prone to modulation [20,21]. Therefore, BDNF emerges as a potential candidate for mediating the switch between acute neuroinflammation (which is beneficial in resolving brain-noxious stimuli) and chronic neuroinflammation, which is thought to be a main player in the progression of neurodegenerative diseases [2].

To date, it has not been explored whether A_2A_R controls microglial levels of neurotrophic factors or its involvement in autocrine/paracrine actions potentially sustaining microgliosis over time. Thus, this study was designed to test whether the A_2A_R blockade modulates BDNF secretion and microglial proliferation induced by the inflammatory trigger lipopolysaccharide (LPS), which would indicate that A_2A_R acts as a key controller of microglial function and neuroinflammation.

## Methods

### Cell line and primary microglial cultures

A murine microglial cell line, N9 (a kind gift from Professor Claudia Verderio, CNR Institute of Neuroscience, Cellular and Molecular Pharmacology, Milan, Italy), was grown in an RPMI medium supplemented with 30 mM glucose (Sigma, Sintra, Portugal), 100 U/mL penicillin and 100 μg/mL streptomycin (GIBCO, Invitrogen, Porto, Portugal).

Primary microglial cultures were prepared as previously described [22]. Briefly, primary cultures of glial cells were obtained from a postnatal (P1-P5) C57BL6 mouse and maintained for 15 days in the DMEM-F12 medium with glutamax (Invitrogen) containing 10% fetal bovine serum (Invitrogen), 0.25% gentamycin (Invitrogen) and 0.25 ng/mL M-CSF (murine-colony stimulating factor, Peprotech, Rocky Hill, New Jersey, USA). Microglia were then separated from the mixed primary culture by shaking (200 rpm for 2 hours), and plated in the DMEM-F12 medium with glutamax containing 0.25% gentamycin (Invitrogen).

Cells were kept at 37°C under a humidified atmosphere with 95% O_2_ and 5% CO_2_. Viable cells (identified by counting trypan-blue-excluding cellular elements) were plated at a density of 5 × 10^5^ cells per cm^2^ in 6 well trays for Western blotting and enzyme-linked immunosorbent assay (ELISA) or 1 × 10^5^ cells per cm^2^ in 12 well trays for proliferation and immunocytochemistry studies.

### Microglial cell pharmacological treatment

In order to evaluate the ability of an inflammatory trigger to control the cellular content of BDNF over time, N9 cells were challenged with 100 ng/mL LPS (from *Escherichia coli*, serotype 055:B5 from Sigma) for 3, 6 and 12 hours. This concentration of LPS was chosen since it was previously shown to induce changes of A_2A_R density in microglial cells [23], which we now aim to pharmacologically manipulate to modulate BDNF secretion/function. Thus, microglial cells were pre-incubated (20 minutes before LPS) with a supra-maximal and selective concentration (50 nM) of an A_2A_R antagonist, SCH58261 [24], which was present until the end of the experiment (3, 6 or 12 hours). Given that we were able to identify the tipping time point of the changes in BDNF levels and A_2A_R modulation effects, subsequent experiments were carried out at this time point (6 hours). In all these experiments, 20 to 30 minutes before adding LPS, N9 cells were incubated with different modulators used in supra-maximal and selective concentrations gauged from our previous experience in different preparations [25,26]: adenosine deaminase (ADA, 1 U/mL, which removes endogenous adenosine; Sigma), H89 (1 μM, a protein kinase A (PKA) inhibitor; Tocris, Madrid, Spain), chelerythrine (6 μM, a protein kinase C (PKC) inhibitor; Calbiochem, Lisbon, Portugal), or anti-human BDNF polyclonal antibody (10 μg/mL; Promega, Lisbon, Portugal), a concentration chosen according to [27]. ADA and the anti-human BDNF polyclonal antibody were directly diluted in the culture medium; H89 and chelerythrine were made up to a 25 mM stock solution in water to dilute in the culture medium.

In the experiments carried out in the absence of LPS, N9 cells were incubated with PKA upstream modulators used in concentrations gauged from our previous experience in different preparations (for example [25,26]), namely: CGS21680 (30 nM; Sigma), an A_2A_R selective agonist; forskolin (1 μM; Ascent Scientific, Cambridge, UK), an activator of adenylate cyclase; 8-Br-AMP (5 μM; Tocris), a cyclic AMP (cAMP) analog, or with exogenously added BDNF (Sigma) used in a concentration (20 ng/mL) able to modulate A_2A_R-mediated neuronal functions [28]. BDNF was directly diluted in the culture medium, whereas CGS21680 and forskolin were made up in dimethyl sulfoxide (100 mM) and 8-Br-AMP was made up in water (100 mM) to dilute in the culture medium. We always tested the impact of the vehicles of the drugs and modulators on each measure and found that all the used vehicles were devoid of effects (data not shown).

### Western blotting

Cell lysates were obtained in a lysis solution containing 150 mM NaCl, 50 mM Tris–HCl, 1 mM ethylenediamine tetraacetic acid (EDTA), 1% NP-40 Igepal (Sigma, Sintra, Portugal), 0.1% sodium dodecyl sulfate (SDS), 0.5% sodium deoxycolate, 1 mM (phenylmethylsulfonyl fluoride (PMSF), 1 mM sodium ortovanadate, 1 mM NaF, 1 μg/mL CLAP (protease inhibitor cocktail; Sigma). After cell scraping for homogenization, the total amount of protein was quantified using the bicinchoninic acid (BCA; Thermo Scientific, Loures, Portugal) method. Samples were then loaded onto gels with 7.5% (to detect A_2A_R, 50 kDa and pro-BDNF, 37.5 kDa) or 15% (to detect mBDNF, 13 kDa) of acrylamide plus bisacrylamide (BioRad, Amadora, Portugal); proteins were separated by electrophoresis (100 to 120 V for 1 hour) using a bicine-buffered solution (20 mM Tris, 192 mM bicine and 0.1% SDS, pH 8.3) and then transferred (300 mV, 100 minutes, 4°C) to polyvinylidene difluoride (PVDF) membranes (0.45 μm pore diameter) (GE Healthcare, Little Chalfont, Buckinghamshire, UK). Blots were then blocked for 1 hour at room temperature (RT) with 5% low-fat milk in Tris-buffered saline (20 mM Tris, 140 mM NaCl, pH 7.6, TBS) with 0.1% Tween 20 (TBS-T) and incubated overnight at 4°C with primary antibodies diluted in TBS-T with 0.5% low-fat milk. The tested primary antibodies were mouse monoclonal anti-BDNF (1:1000; Sigma) and mouse anti-A_2A_R (1:1000; Millipore, Lisbon, Portugal). After rinsing three times with 0.5% low-fat milk in TBS-T, membranes were incubated for 1 hour at RT with alkaline phosphatase-conjugated secondary antibodies (1:2000; Amersham, Piscataway, New Jersey, USA). Protein immunoreactive bands were visualized in a Versa Doc Imaging System (Model 3000, BioRad Laboratories), after the incubation of the membrane with enhanced chemofluorescence reagent (ECF; GE Healthcare).

Re-probing of the same membranes with a different antibody was achieved by washing the ECF in 40% methanol for 30 minutes and stripping the previous antibodies in a solution of 0.2 M glycine with 0.1% SDS and 1% (v/v) Tween 20, pH 2.2, for 1 hour. After washing (3 times with TBS-T for 20 minutes), membranes were blocked and incubated with primary and respective secondary antibodies, namely for mouse anti-α-tubulin (1:20000; Sigma) to confirm that similar amounts of sample were loaded to the different lanes.

### Immunocytochemistry

Cells were fixed with 4% paraformaldehyde (Sigma) and permeabilized for 20 minutes in 0.25% Triton X-100 (Sigma) in a phosphate-buffered saline (PBS) solution (137 mM NaCl, 2.7 mM KCl, 10 mM NaH_2_PO_4_, 1.8 mM KH_2_PO_4_, pH 7.4). Unspecific binding was prevented by incubating cells in PBS with 3% bovine serum albumin (BSA) and 5% normal horse serum. Cells were incubated overnight at 4°C in PBS with 3% BSA and 5% normal horse serum and including the primary antibody (mouse anti-A_2A_, 1:250; Millipore). Cells were then washed and incubated for 1 hour at RT with an Alexa Fluor 488 donkey anti-mouse (1:400; Molecular Probes, Lisbon, Portugal) secondary antibody. Membrane ruffling, which is characteristic of activated microglia, was probed using a marker for filamentous actin, phalloidin, by incubating cells for 20 minutes at RT with PBS containing actin-stain 670 fluorescent phalloidin (1:75; Cytoskeleton, Denver, USA). For nuclear labeling, N9 cells were stained with DAPI (0.1 mg/mL; Invitrogen). The coverslips were mounted in the Prolong Gold Antifade fluorescent medium (Invitrogen). In order to check for non-specific labeling of the secondary antibodies or cross-reactivity between secondary antibodies, staining was tested in the absence of each primary antibody and in the absence of both primary antibodies. Fluorescent images were acquired using a Zeiss Imager Z2 fluorescence microscope equipped with an AxioCam HRm and 63x Plan-ApoChromat oil objective (1.4 numerical aperture), with Axiovision SE64 4.8.2 software. Ten images were randomly taken from each coverslip (three per condition in each experiment).

### BrdU incorporation assay

Microglial proliferation was evaluated by measuring the incorporation of 5-bromo-2'-deoxyuridine (BrdU; Sigma), a synthetic nucleoside that can be incorporated into newly synthesized DNA, replacing thymidine during cell replication. Cells were incubated with BrdU (10 μM) for the last 2 hours of pharmacological treatment, fixed in 4% paraformaldehyde, washed in TBS with 0.3% Triton X-100 and maintained in 1 M HCl at 37°C for 30 minutes. Non-specific binding was prevented by incubation for 1 hour in TBS with 3% BSA and 1% Triton X-100. Cells were incubated overnight at 4°C with a primary rat antibody anti-BrdU (1:100; Serotec, Oxford, UK) in a 0.1% Triton X-100 and 0.3% BSA solution, washed and incubated for 2 hours at RT with an Alexa Fluor 594 donkey anti-rat secondary antibody (1:200; Molecular Probes). For nuclear staining, cells were incubated for 5 minutes at RT with Hoechst 33342 (10 μg/mL; Molecular Probes) in 0.3% BSA, and mounted in Dakocytomation fluorescent medium (Dakocytomation Inc., California, USA). Fluorescent images were acquired using an Axioskop 2 Plus fluorescence microscope (Zeiss, PG-Hitec, Lisbon, Portugal). The number of proliferating cells (BrdU-positive) was counted and expressed as a percentage of the total cells stained with Hoechst 33342 [29].

### BDNF enzyme-linked immunosorbent assay

The extracellular levels of BDNF were measured in the supernatant of N9 cells using the BDNF Emax Immunoassay System (Promega, Madison, USA). Each well of a 96-well polystyrene plate was incubated overnight at 4°C with 80 μL of anti-BDNF monoclonal antibody (1:1000) in coating carbonate buffer (50 mM, pH 9.7). Non-adsorbed antibody was discarded and rinsed off by washing once in a TBS-T buffer (20 mM Tris–HCl, pH 7.6, 150 mM NaCl and 0.05% (v/v) Tween 20). Unspecific binding was prevented by blocking with 80 μL Promega 1x Block and Sample Buffer (BB 1x) for 1 hour at RT. Plates were then washed (as mentioned before) and 80 μL of each sample or standard (7.8 pg/mL to 500 pg/mL) were loaded in triplicate to the plates (2 hours with 300 rpm shaking at RT). After washing (5 times in TBS-T wash buffer), 80 μL of anti-human BDNF polyclonal antibody (1:500 in BB 1x) was added to each well and the plates were incubated at RT (2 hours with 300 rpm shaking). After washing (5 times in TBS-T wash buffer), the plates were incubated for 1 hour with shaking (300 rpm) with 80 μL anti-IgY horseradish peroxidase conjugate (1:200 in BB 1x). After the last wash with the TBS-T buffer, 80 μl of TMB One solution was used as developer and the reaction was stopped by adding 80 μL of HCl 1 M. Absorbance was measured at 450 nm. BDNF levels are reported as pg/mL normalized per total amount of protein.

### Data analysis

Values are presented as mean ± standard error of the mean (SEM) of *n* experiments. Either a Student’s *t* test for independent means or a one-way analysis of variance (ANOVA) followed by a Newman–Keuls *post hoc* test, was used to define statistical differences between absolute values, which were considered significant at *P* < 0.05 unless otherwise specified. Note that although the impact of several drugs and modulators are presented as percentage values for the sake of clarity, the statistical comparisons were always carried out using the absolute values.

## Results

### LPS induces time-dependent changes in BDNF cellular levels, an effect dependent upon adenosine A_2A_ receptor tonic activation

To explore the ability of A_2A_R to modulate BDNF levels in microglial cells in response to an inflammatory stimulus, we used a microglial cell line (N9), which has been successfully used previously to dissect classical microglial responses (secretion of inflammatory mediators, microglial proliferation and phagocytosis) in inflammatory-like conditions [30,31]. N9 cells were activated with LPS, a component of the Gram-negative bacteria cell membrane, which is a well-characterized inflammatory stimulus able to induce microglial activation and the subsequent secretion of trophic factors, including BDNF (for example [9,12]).

**Figure 1:**
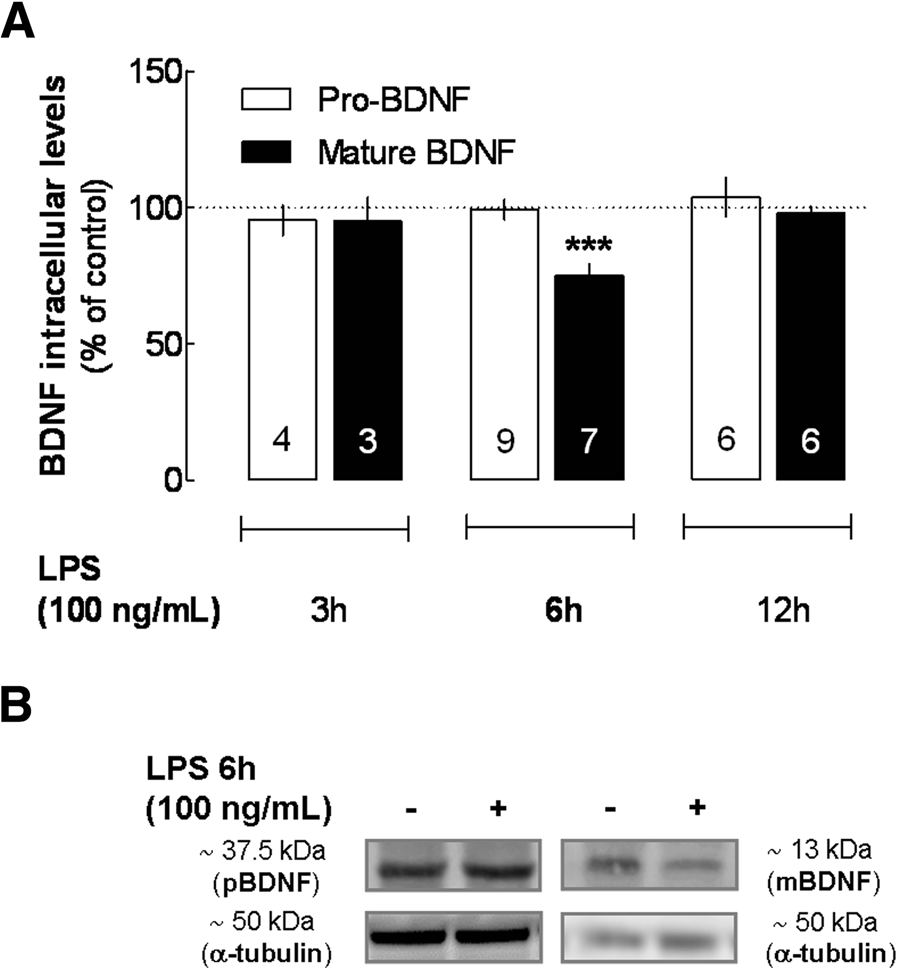
**Effect of LPS on the intracellular levels of pro- and mature BDNF in murine N9 microglial cells. (A)** N9 cells were exposed to LPS (100 ng/mL) for 3, 6 and 12 hours, then lysed and homogenized for Western blot analysis of pro- (open bars) and mature BDNF (filled bars) immunoreactivities (37.5 and 13 kDa, respectively). Results are expressed as mean ± SEM of *n* (as indicated in each bar) independent experiments (*** *P* < 0.001, compared with non-treated cells, using the Newman–Keuls multiple comparison test) and 100% represents the pro- and mBDNF in cells that were not exposed to LPS. **(B)** Representative blot of the LPS (100 ng/mL for 6 hours)-mediated decrease of intracellular mature BDNF and its inability to interfere with the intracellular levels of pro-BDNF; the blots compare BDNF immunoreactivity from cells exposed (+) or not (−) to LPS. BDNF, brain-derived neurotrophic factor; LPS, lipopolysaccharide; mBDNF, mature protein BDNF; SEM, standard error of the mean.

We began testing the time course (3 up to 12 hours) of the impact of LPS (100 ng/mL) on the intracellular levels of BDNF. BDNF is synthesized as a precursor protein (pro-BDNF), which is subsequently cleaved intra- and/or extracellularly (by peptidases and convertases) to form the mature protein (mBDNF) [19,32,33], which can be detected by Western blot analysis (37.5 and 13 kDa, respectively). In the present experimental conditions, LPS (100 ng/mL) did not affect the cellular levels of pro-BDNF at any given time point (Figure 1A,B, *P* > 0.05 compared with non-treated cells). However, 6 hours of exposure to LPS induced a decrease of the cellular levels of the mature protein (74.9 ± 4.3%, *n* = 7, *P* < 0.001 compared with non-treated cells), which returned to values similar to those observed in non-treated cells 6 hours later, that is, at 12 hours of exposure to LPS (Figure 1A,B).

**Figure 2:**
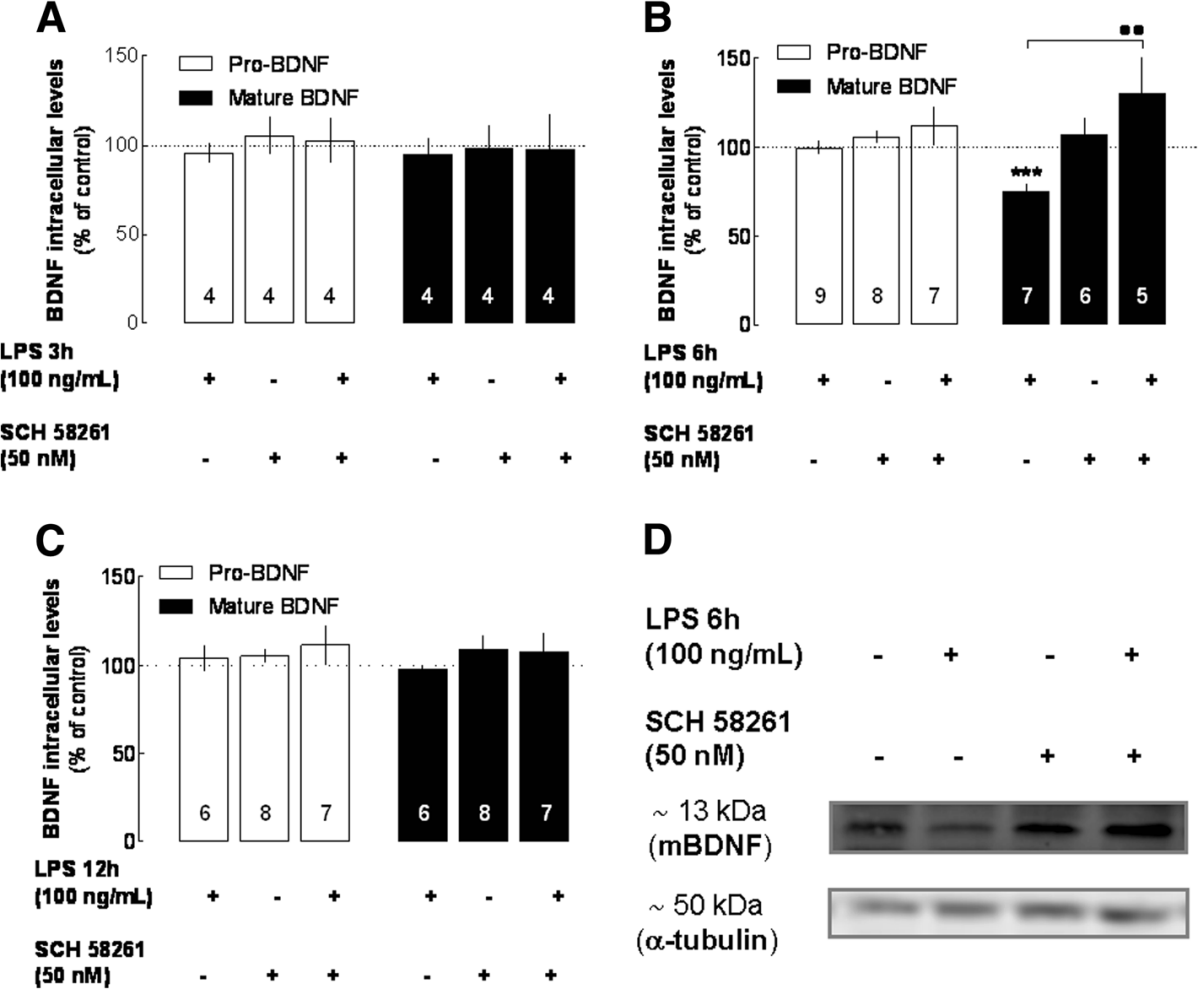
**Effect of LPS on the intracellular levels of pro- and mature BDNF in the presence of a selective A**_**2A**_**R antagonist in murine N9 microglial cells.** The A_2A_R antagonist SCH58261 (50 nM) prevented the LPS (100 ng/mL)-induced modification of mature BDNF levels selectively at 6 hours **(B)**. In contrast, the A_2A_R blockade failed to affect the levels of pro- and mature BDNF at 3 hours **(A)** or 12 hours **(C)** of LPS exposure. Note that A_2A_R only affected BDNF levels when they were challenged with LPS. Results are expressed as mean ± SEM of *n* (as indicated in each bar) independent experiments (*** *P* < 0.001, compared with non-treated cells; ·· *P* < 0.01, compared with LPS-treated cells, using the Newman–Keuls multiple comparison test) and 100% represents the pro- and mBDNF in cells that were not exposed to LPS. **(D)** Representative blot of the modulation by SCH58261 of intracellular mature BDNF in cells challenged for 6 hours with LPS. We verified (not shown) that none of the vehicles of the tested drugs (water or dimethyl sulfoxide) modified BDNF levels. A_2A_R, A_2A_ receptor; BDNF, brain-derived neurotrophic factor; LPS, lipopolysaccharide; mBDNF, mature protein BDNF; SEM, standard error of the mean.

We have previously reported that LPS is able to induce microgliosis and the production of pro-inflammatory mediators under A_2A_R control [9]. In parallel, it is known that A_2A_R modulates BDNF levels both in neurons [34] and in native tissue [35]. Since this effect has not yet been reported in microglia, we tested the ability of a supra-maximal and selective concentration (50 nM) of an A_2A_R antagonist, SCH58261 [24], to control BDNF levels in the absence and in the presence of LPS. As shown in Figure 2, the A_2A_R antagonist prevented the LPS-induced decrease of mBDNF at 6 hours (129.5 ± 23.04%, *n* = 5, *P* < 0.01 compared with LPS-treated cells, Figures 2B,D), whereas SCH58261 was devoid of effects on BDNF levels either when LPS has no effects (that is*,* at 3 or 12 hours; Figure 2A,C) or in the absence of LPS (*P* > 0.05 compared with non-treated cells or LPS-treated cells for 3 and 12 hours).

### LPS-induced decrease of BDNF cellular levels is related to an increase of BDNF secretion, an effect dependent upon a tonic activation of A_2A_R

**Figure 3:**
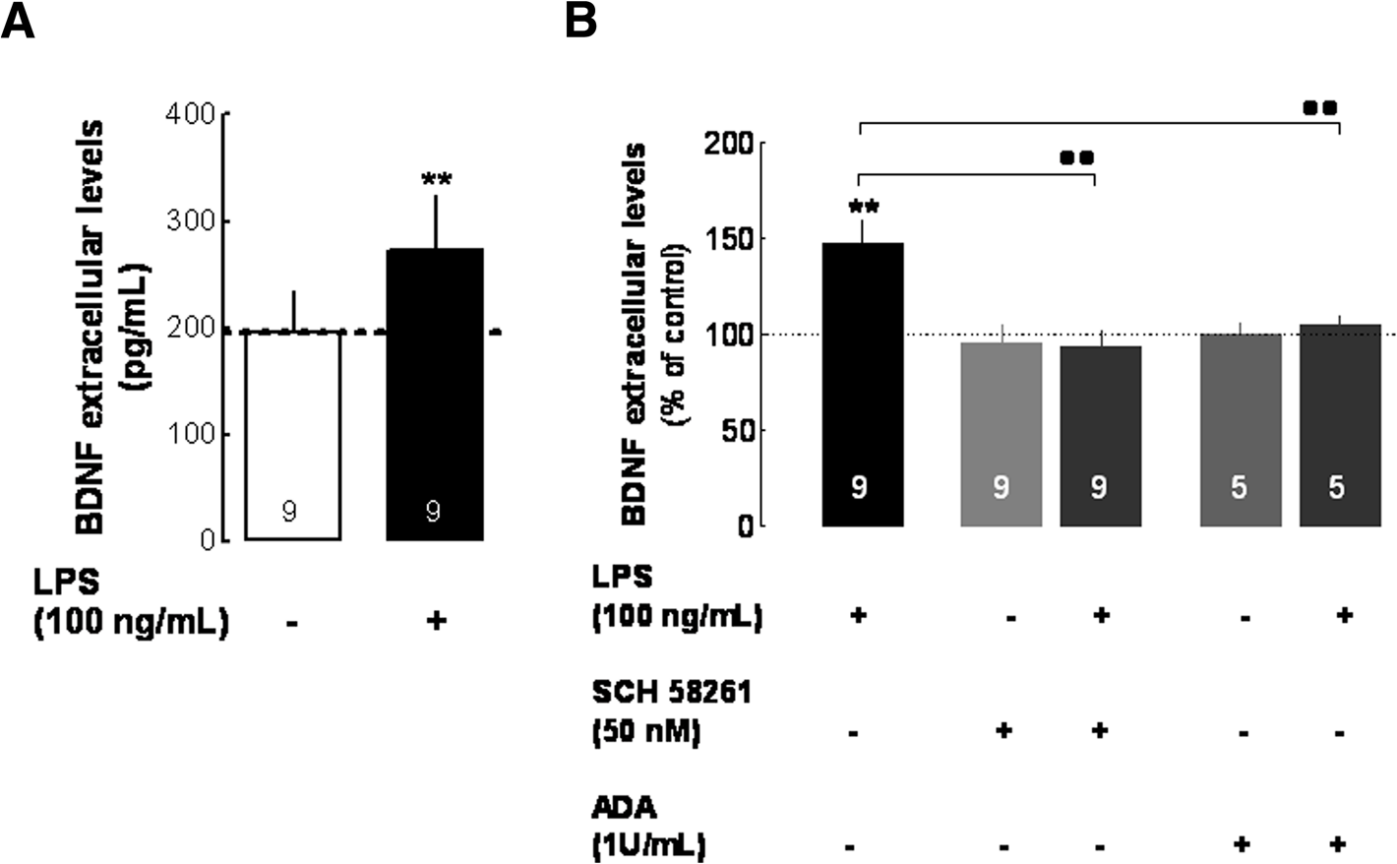
**Endogenous extracellular adenosine, through activation of A**_**2A**_**R, mediates the LPS-induced enhancement of BDNF secretion from microglial N9 cells.** Cells were incubated with LPS (100 ng/mL for 6 hours) in the absence or in the presence of the A_2A_R antagonist, SCH58261 (50 nM) or in the absence or presence of adenosine deaminase (ADA, 1 U/mL), which converts adenosine into its inactive metabolite, inosine. The incubation medium was collected to quantify the extracellular free mature BDNF by ELISA. **(A)** Comparison between absolute values of BDNF (pg/mL) in the absence and in the presence of LPS. **(B)** LPS increased the extracellular levels of BDNF, an effect prevented by A_2A_R antagonism and by adenosine removal from the medium with ADA (percentage of effect; 100% represents BDNF levels in cells that were not exposed to LPS). Results are expressed as mean ± SEM of *n* (as indicated in each bar) independent experiments (** *P* < 0.01, compared with non-treated cells; ·· *P* < 0.01, compared with LPS-treated cells, using the Newman–Keuls multiple comparison test). We verified (not shown) that none of the vehicles of the tested drugs (water or dimethyl sulfoxide) modified BDNF levels. A_2A_R, A_2A_ receptor; ADA, adenosine deaminase; BDNF, brain-derived neurotrophic factor; LPS, lipopolysaccharide; SEM, standard error of the mean.

The LPS-induced decrease of mBDNF at 6 hours of exposure may be explained by changes in BDNF expression and/or secretion. The fact that LPS did not influence the levels of pro-BDNF (see Figures 1 and 2) suggests that changes in secretion are more likely to be involved. Thus, we tested the influence of LPS upon BDNF secretion by measuring free mature BDNF levels in the culture medium, as assessed by ELISA. Endogenous levels of BDNF in non-treated cells (196.2 ± 37.6 pg/mL, *n* = 9, Figure 3A) were increased by 147.3 ± 12% (*n* = 9, *P* < 0.01) in the presence of LPS (100 ng/mL for 6 hours), reaching extracellular values of 272.1 ± 51.3 pg/mL (Figure 3A,B). The A_2A_R antagonist, SCH58261 (50 nM), notably prevented this LPS-induced BDNF secretion (93.6 ± 8.7%, *n* = 9, *P* < 0.01 compared with LPS-treated cells, Figure 3B). These results indicate that A_2A_R activation by endogenous adenosine is required to allow the LPS-induced secretion of BDNF. Accordingly, the presence of ADA (1 U/mL), which removes endogenous adenosine thus impairing A_2A_R activation, also prevented the effect of LPS upon BDNF release (104.7 ± 4.2%, *n* = 5, *P* < 0.01 compared with LPS-treated cells, Figure 3B).

### A_2A_R control the LPS-induced secretion of BDNF through the cAMP-PKA pathway

**Figure 4:**
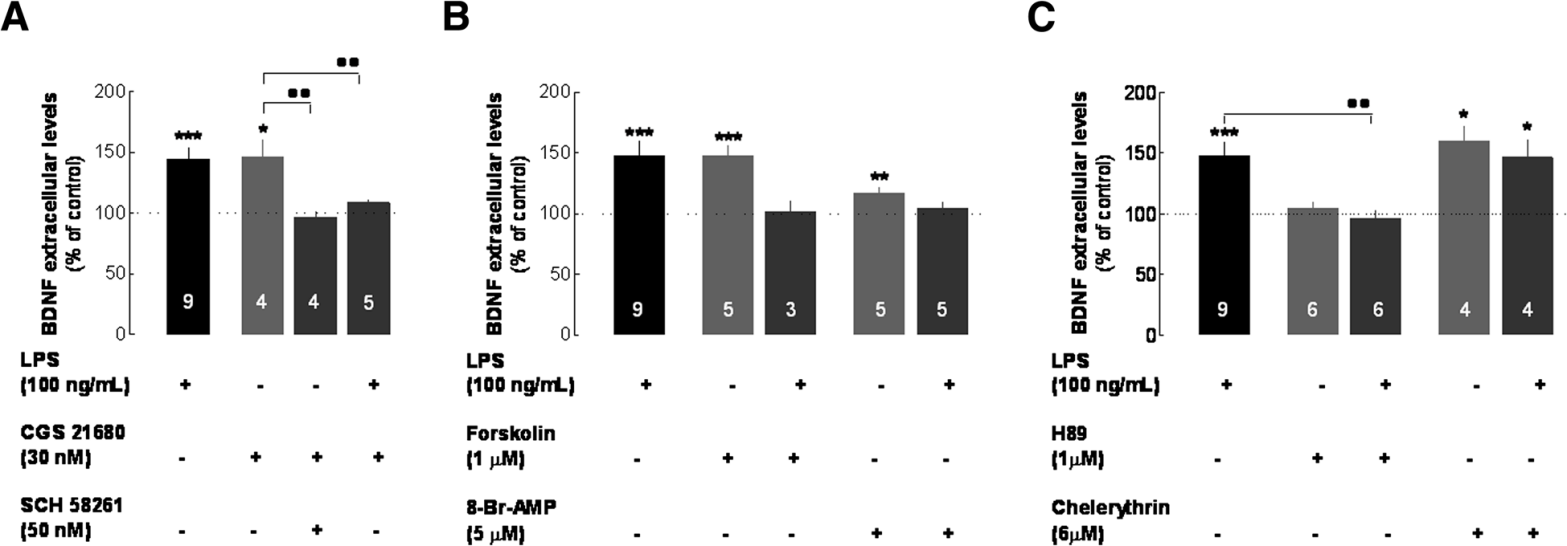
**The LPS-induced enhancement of BDNF secretion mediated by A**_**2A**_**R involves the recruitment of the AC-cAMP-PKA transducing pathway, whereas PKC controls the constitutive release of BDNF from N9 microglial cells.** In all experiments, the medium was collected after 6 hours for quantitative analysis of extracellular BDNF (ELISA). **(A)** Cells were incubated with the A_2A_R agonist CGS21680 (30 nM) in the absence or in the presence of the A_2A_R antagonist SCH58261 (50 nM) or LPS (100 ng/mL). **(B)** Cells were incubated with the adenylyl cyclase (AC) activator, forskolin (1 μM) or with the cAMP analog, 8-Br-cAMP (5 μM) in the absence and in the presence of LPS (100 ng/mL). **(C)** Cells were incubated with the PKA inhibitor, H89 (1 μM) or with the PKC inhibitor, chelerythrine (6 μM) in the absence and in the presence of LPS (100 ng/mL). Results are expressed as mean ± SEM of *n* (as indicated in each bar) independent experiments (*** *P* < 0.001, ** *P* < 0.01, * *P* < 0.05, compared with non-treated cells; ·· *P* < 0.01, compared with LPS-treated cells, using the Newman–Keuls multiple comparison test) and 100% represents the pro- and mBDNF in cells that were not exposed to LPS. We verified (not shown) that none of the vehicles of the tested drugs (water or dimethyl sulfoxide) modified BDNF levels. A_2A_R, A_2A_ receptor; ADA, adenosine deaminase; BDNF, brain-derived neurotrophic factor; cAMP, cyclic AMP; LPS, lipopolysaccharide; PKA, protein kinase A; PKC, protein kinase C; SEM, standard error of the mean.

The key role of A_2A_R in mediating the LPS-induced secretion of BDNF suggests that the direct activation of A_2A_R should be able to trigger the release of BDNF from microglial N9 cells. Indeed, the selective A_2A_R agonist, CGS21680 (30 nM), enhanced *per se* (that is*,* in the absence of LPS) the secretion of BDNF (146.2 ± 14.3%, *n* = 4, *P* < 0.05 compared with non-treated cells) to an extent similar to the effect of LPS (*P* > 0.05 compared with LPS-treated cells), an effect prevented by the A_2A_R antagonist SCH58261 (50 nM) (96.3 ± 10%, *n* = 4, *P* < 0.01 compared with CGS21680-treated cells) (Figure 4A).

A_2A_R are G protein-coupled receptors and most of their effects involve AC activation and the subsequent raise in cAMP levels, which activate PKA (for a review, see, for example, [36]). Accordingly, adenosine modulates peripheral immune responses through the activation of A_2A_R and the recruitment of the cAMP-PKA pathway [6]. It has previously been reported that PKA notably plays a crucial role in the control of the depolarization-evoked release of BDNF from neurons [20]. Thus, we tested to see if the AC-cAMP-PKA pathway would control BDNF secretion from microglial cells. As shown in Figure 4B, the activation of AC with forskolin (1 μM) or the cAMP analog 8-bromo-cAMP (5 μM), mimicked the effect of LPS, triggering BDNF secretion (147.4 ± 9.1%, *n* = 5, *P* < 0.001 compared with non-treated cells and 120.9 ± 3.4%, *n* = 5, *P* < 0.01 compared with non-treated cells) to an extent similar to that caused by LPS (both have *P* > 0.05 compared with LPS-treated cells). Compatible with this scenario, we report that the PKA inhibitor H89 (1 μM) prevented the effect of LPS upon BDNF secretion (95.8 ± 7.7%, *n* = 6, *P* < 0.01 compared with LPS-treated cells, Figure 4C), further suggesting that the tonic activation by endogenous adenosine of A_2A_R operating through the cAMP-PKA pathway underlies the ability of LPS to trigger BDNF secretion.

Notably, we observed that H89 failed to modify the basal outflow of BDNF from microglial N9 cells (Figure 4C), indicating that the AC-cAMP-PKA pathway can be recruited by A_2A_R to bolster the release of BDNF upon LPS-induced microgliosis, but does not mediate the constitutive release of BDNF. In contrast, the PKC inhibitor, chelerythrine (6 μM), enhanced the constitutive outflow of BDNF (160.3 ± 12.3%, *n* = 4, *P* < 0.05 compared with non-treated cells; Figure 4C), but failed to affect the LPS-induced secretion of BDNF (146.4 ± 15.0%, *n* = 4, *P* > 0.05 compared with LPS-treated cells; Figure 4C). This indicates that the transducing pathways involved in the constitutive and LPS-induced release of BDNF are different and that the LPS-induced enhancement of BDNF secretion selectively recruits the A_2A_R operating the cAMP-PKA pathway.

In accordance with our working hypothesis that the LPS-induced release of BDNF requires the recruitment of A_2A_R and the activation of the AC-cAMP pathway, we expected to record an ability for CGS21680 (an activator of A_2A_R), forskolin (an activator of AC) or 8-Br-cAMP to occlude the ability of LPS to enhance BDNF release. Surprisingly, we observed that the activation of the A_2A_R-AC-cAMP system before the LPS challenge actually changed the set-up of the N9 cells to such an extent that the simultaneous presence of LPS and any of the activators of the A_2A_R-AC-cAMP axis (30 nM CGS21680, 1 μM forskolin or 5 μM 8-Br-cAMP) now failed to modify (*P* > 0.05) the release of BDNF compared to the control (Figures 4A,B). This prompts the hypothesis that the LPS signaling pathway or the ability to release BDNF might be affected by this pre-exposure to these activators of the A_2A_R-AC-cAMP axis, a question that warrants further detailed mechanistic investigation.

**Figure 5:**
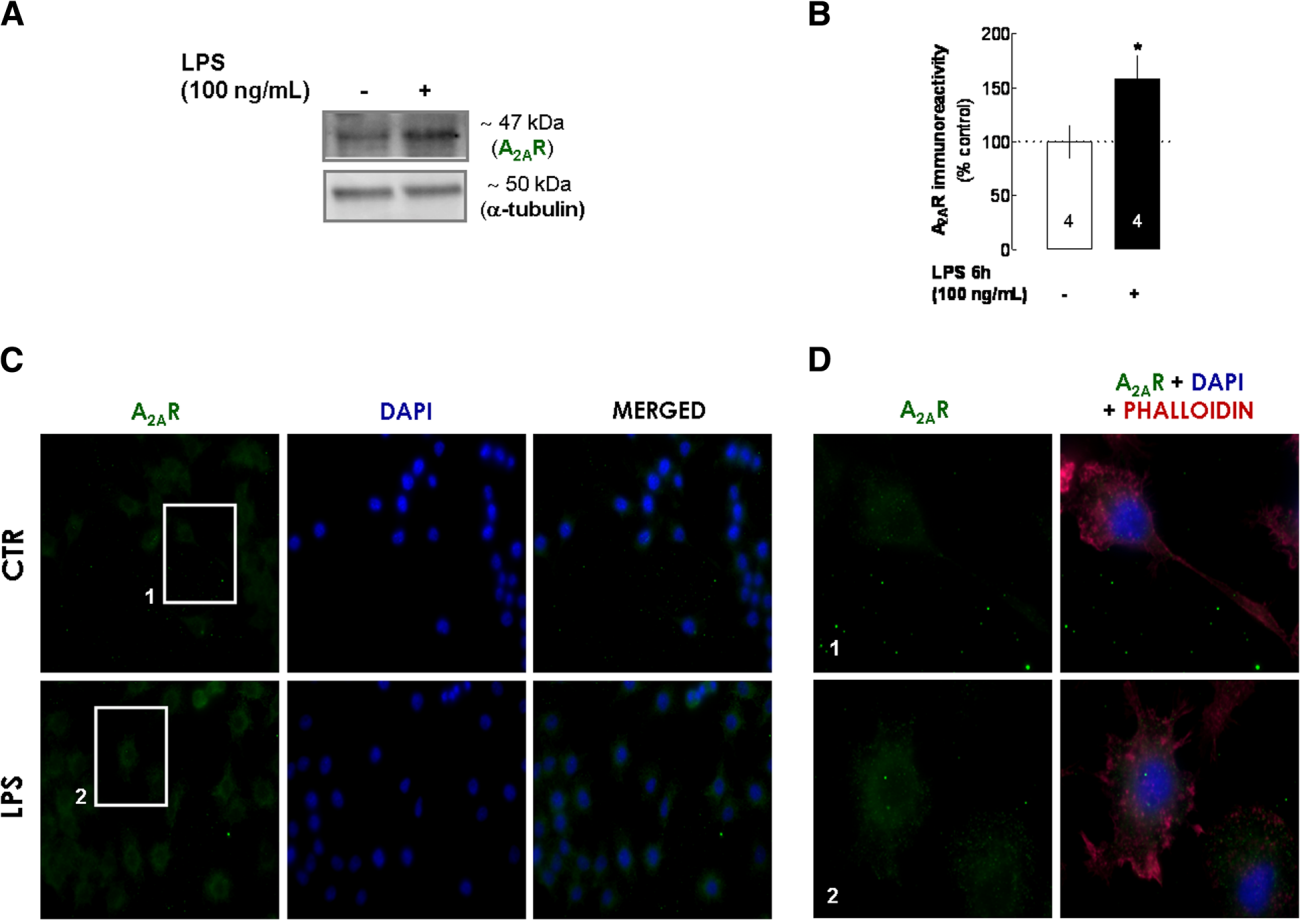
**Effect of LPS on the density of A**_**2A**_**R in N9 microglial cells. (A)** Representative blot of the LPS (100 ng/mL for 6 hours)-mediated increase of A_2A_R density; the blot compares A_2A_R immunoreactivity from cells exposed (+) or not (−) to LPS in four independent experiments. **(B)** Cells were exposed to LPS (100 ng/mL for 6 hours) and then lysed and homogenized for Western blot analysis. Quantitative analysis of A_2A_R immunoreactivity in the presence of LPS was compared with non-treated cells (taken as 100%). Results are expressed as mean ± SEM of *n* (as indicated in each bar) independent experiments (* *P* < 0.05, compared with non-treated cells, using Student’s *t* test). **(C)** Immunocytochemistry confirmed the LPS-induced increase in A_2A_R immunoreactivity (representative images of independent experiments). Double-labeling for A_2A_R (green) and for the structural protein, phalloidin (far red), was performed in all experiments; phalloidin-labeling is not shown to allow a clear visualization of A_2A_R immunoreactivity (the nuclei were labeled with DAPI, in blue). **(D)** Magnification of cellular elements 1 (microglia in basal conditions) and 2 (activated cell) in (**C**), showing in detail the increase in A_2A_R density in activated cells (phalloidin-labeling is shown to allow the visualization of LPS-induced morphological changes of microglial cells). A_2A_R, A_2A_ receptor; LPS, lipopolysaccharide; SEM, standard error of the mean.

This different role for A_2A_R in the control of LPS-induced enhancement of BDNF secretion (LPS requires the subsequent activation of A_2A_R, whereas the pre-activation of A_2A_R inhibited the LPS-induced release of BDNF) led us to test the hypothesis that LPS might up-regulate A_2A_R to bolster BDNF secretion from N9 microglial cells. As shown in Figure 5, both Western blot (Figure 5A,B) and immunocytochemical (Figure 5C) analysis of N9 cells revealed an enhancement of A_2A_R immunoreactivity at the same time point where LPS-induced enhancement of BDNF secretion was observed (that is*,* at 6 hours). The temporal correlation between the enhancement of A_2A_R density and the PKA-dependent increase of BDNF secretion suggests that LPS-induced PKC-PKA shifting in the regulation of BDNF secretion may be triggered by the increased A_2A_R signaling subsequent to an increase in receptor density and activation.

### LPS promotes microglial proliferation, an effect dependent upon BDNF and A_2A_R tonic activation

**Figure 6:**
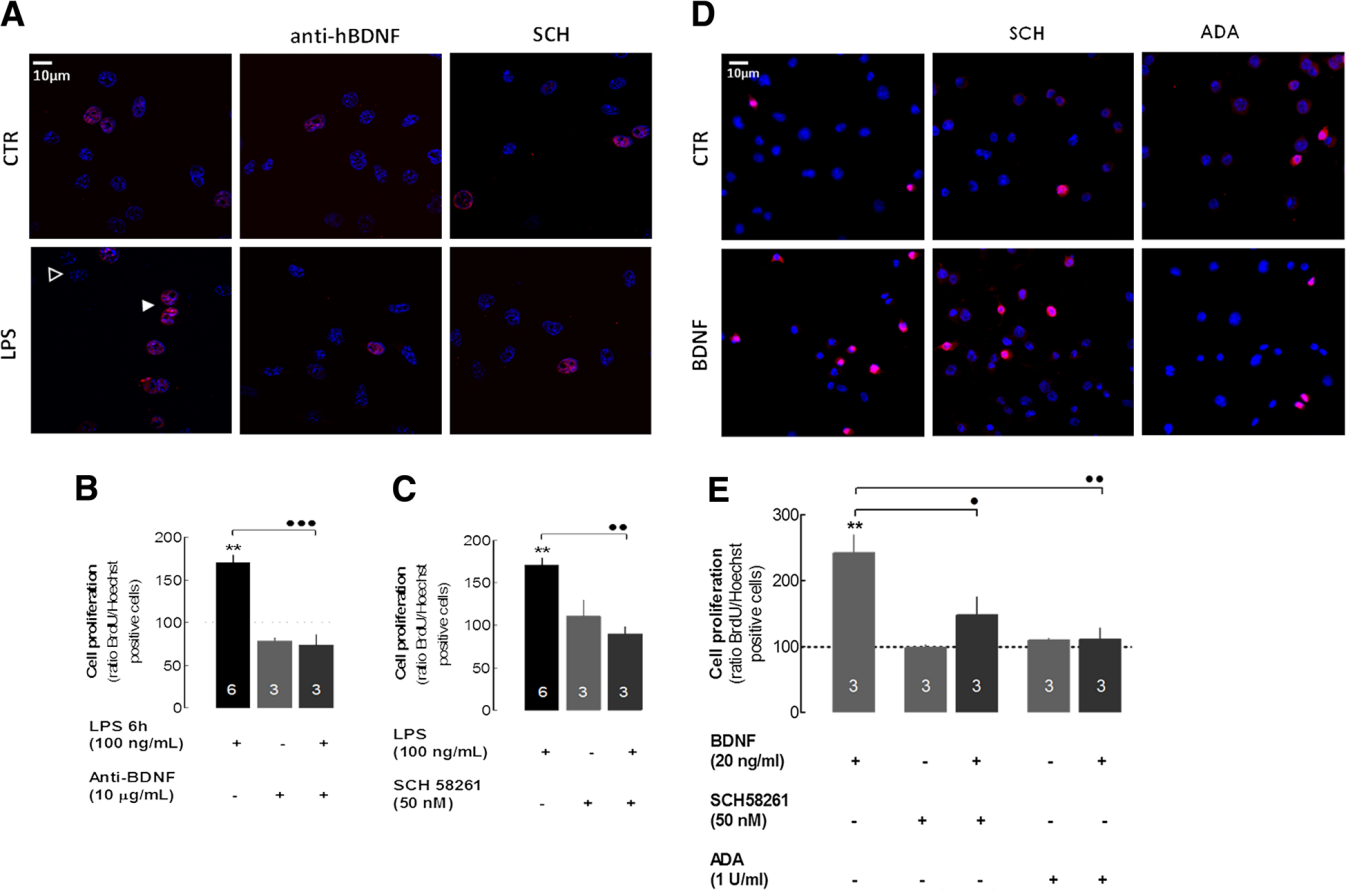
**LPS triggers microglial N9 proliferation in a manner dependent on extracellular BDNF and A**_**2A**_**R activation.** Cells were exposed to LPS (100 ng/mL) for 6 hours in the presence of BrdU for the last 2 hours. Proliferation was quantified as the number of BrdU-labeled nuclei (red) and expressed as a percentage of the total number of DAPI-labeled nuclei (blue). **(A)** Representative images illustrating the ability of LPS to enhance N9 microglial cell proliferation, an effect prevented both by an anti-BDNF antibody (10 μg/mL) and by the selective A_2A_R antagonist, SCH58261 (50 nM). Average quantitative analysis shows that anti-BDNF antibody **(B)** or SCH58261 **(C)** prevents LPS-induced proliferation. **(D)** Representative images illustrating the ability of added BDNF (20 ng/mL) to enhance N9 microglial cells proliferation, an effect prevented both by the selective A_2A_R antagonist, SCH58261 (50 nM) and by adenosine deaminase (ADA, 1 U/mL). Average quantitative analysis shows the ability of SCH58261 (50 nM) and ADA (1 U/mL) to prevent BDNF effects **(E)**. Results are expressed as mean ± SEM of *n* (as indicated in each bar) independent experiments (** *P* < 0.01, compared with non-treated cells; ··· *P* < 0.001, ·· *P* < 0.01, compared with LPS-treated cells; ·· *P* < 0.01, · *P* < 0.05 compared with BDNF-treated cells using the Newman–Keuls multiple comparison test) and 100% represents proliferation of cells that were not exposed to LPS. A_2A_R, A_2A_ receptor; ADA, adenosine deaminase; BDNF, brain-derived neurotrophic factor; LPS, lipopolysaccharide; SEM, standard error of the mean.

Given that both BDNF and A_2A_R activation promote neuroinflammation and A_2A_R was previously proposed to be required for microglial proliferation [37], we next aimed to extend understanding of the tight interplay between BDNF and A_2A_R in the context of the control of microglial proliferation, a key process for sustaining neuroinflammation. We posited that one of the functions of BDNF released under LPS stimulation of microglial cells might be to promote their proliferation in order to ‘feed’ microgliosis, an effect which might also be controlled by A_2A_R. To begin probing this hypothesis, we first tested the impact of an antibody that specifically recognizes and sequesters BDNF on the LPS-induced proliferation of N9 microglial cells. As anticipated, LPS (100 ng/mL for 6 hours) induced a clear proliferation of N9 microglial cells (170.5 ± 9.2%, *n* = 6, *P* < 0.01 compared with non-treated cells). Notably, this effect was prevented when the cells were simultaneously incubated with the antibody anti-BDNF (79.4 ± 3.5%, *n* = 3, *P* < 0.001 compared with LPS-treated cells; Figure 6A,B). Furthermore, in line with the ability of A_2A_R to control LPS-induced BDNF secretion, we observed that the A_2A_R antagonist, SCH58261 (50 nM), prevented the LPS-induced increase of N9 microglial cell proliferation (90.4 ± 8.1%, *n* = 3, *P* < 0.01 compared with LPS-treated cells, Figure 6A,C).

In order to disentangle whether the A_2A_R blockade was only preventing BDNF secretion or also the impact of BDNF on microglial proliferation, we then evaluated the ability of exogenously added BDNF to directly trigger the proliferation of N9 microglial cells, as well as the ability of SCH58261 to modulate this eventual effect. BDNF (20 ng/mL) increased the proliferation of N9 microglial cells (242 ± 48%, *n* = 3, *P* < 0.01 compared with non-treated cells), an effect prevented by the antibody anti-BDNF (104.3 ± 4.1%, *n* = 3, *P* < 0.05 compared with BDNF-treated cells, data not shown) and by the A_2A_R blockade (147.7 ± 47%, *n* = 3, *P* < 0.05 compared with BDNF-treated cells) or removal of endogenous adenosine with 1 U/mL of ADA (111.2 ± 29%, *n* = 3, *P* < 0.01 compared with BDNF-treated cells, Figure 6D,E).

**Figure 7:**
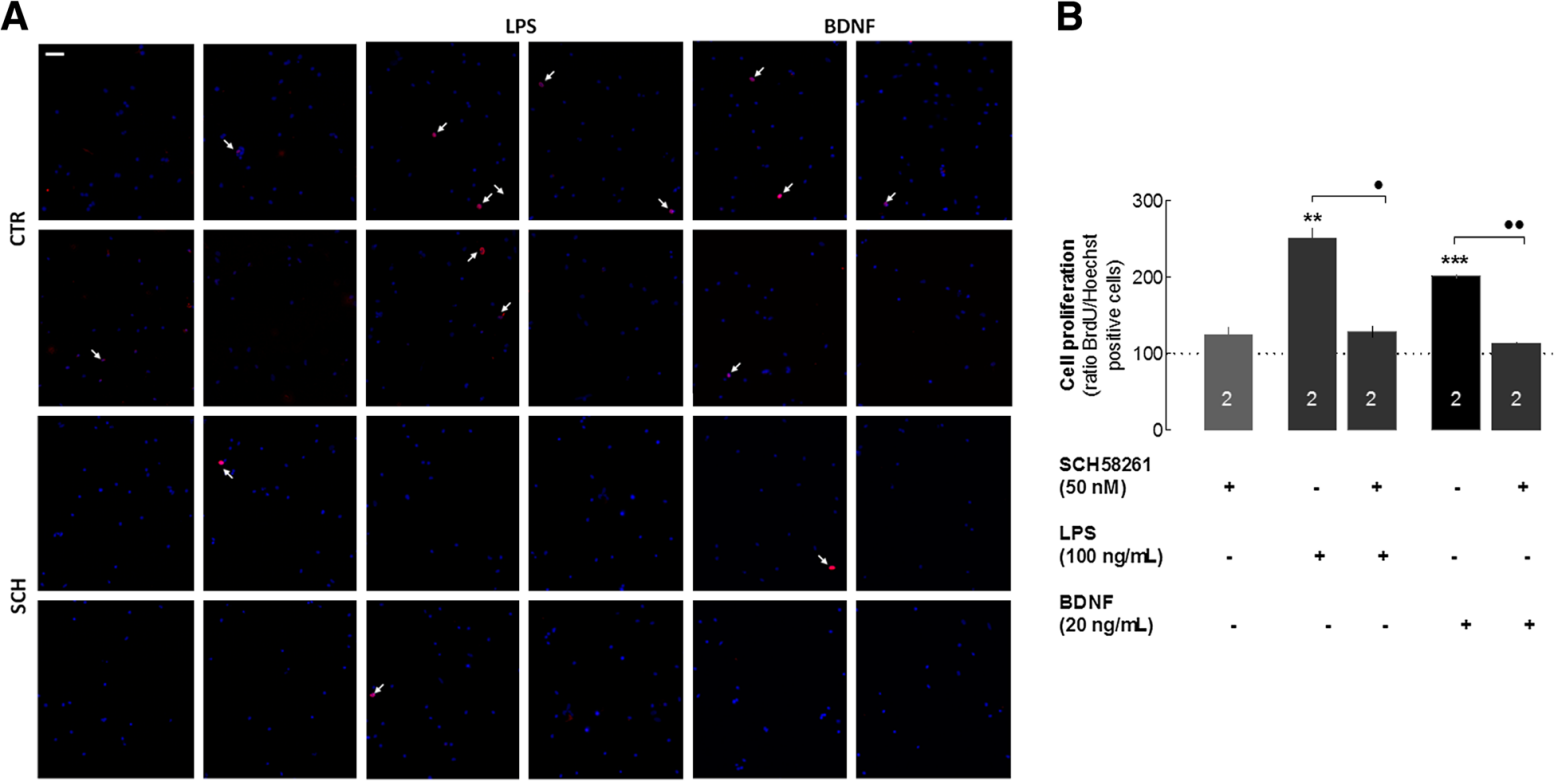
**LPS and BDNF trigger primary microglial proliferation in a manner dependent on A**_**2A**_**R activation.** Cells were exposed to LPS (100 ng/mL) for 6 hours in the presence of BrdU for the last 2 hours. Proliferation was quantified as the number of BrdU-labeled nuclei (red) and expressed as a percentage of the total number of DAPI-labeled nuclei (blue). **(A)** Representative images illustrating the ability of LPS and BDNF to enhance primary microglial proliferation, an effect prevented by the selective A_2A_R antagonist, SCH58261 (50 nM) **(B)**. Results are expressed as mean ± SEM of two independent experiments (** *P* < 0.01, *** *P* < 0.001, compared with non-treated cells; ·· *P* < 0.01, · *P* < 0.05, compared with LPS-treated cells) and 100% represents proliferation of cells that were not exposed to LPS. A_2A_R, A_2A_ receptor; ADA, adenosine deaminase; BDNF, brain-derived neurotrophic factor; BrdU, 5-bromo-2'-deoxyuridine; LPS, lipopolysaccharide; SEM, standard error of the mean.

Finally, we attempted to extend the A_2A_R-mediated control of BDNF-induced proliferation from the context of N9 microglial cells to that of mouse microglia. These mouse primary cultures of microglia (*n* = 2 in triplicate) were exposed to LPS (100 ng/mL) or to BDNF (20 ng/mL) for 6 hours: both LPS (251.1 ± 12.8%, *P* < 0.01 compared with non-treated cells, Figure 7A,B) and BDNF (201.1 ± 2.6%,*P* < 0.05 compared with non-treated cells, Figure 7A,B) increased primary microglial proliferation, an effect prevented by the A_2A_R blockade with SCH58261 (50 nM).

## Discussion

The present study suggest two major conclusions: 1) the activation of microglial cells with LPS triggers a release of BDNF, which plays a key autocrine or paracrine role by stimulating microglial proliferation; 2) both the LPS-induced release of BDNF as well as the resulting proliferation of microglia is prevented by the blockade of adenosine A_2A_R.

In response to noxious stimuli, microglia become activated by changing their morphology, proliferating and releasing molecules, some considered to be neurotoxic and others neuroprotective [1]. Irrespective of the nature of the released substances, microglial responses in the central nervous system must be tightly regulated since the sustained activation of microglia results in a chronic neuroinflammatory process, which is involved in neurodegeneration. Neurotrophic factors, namely BDNF, which are produced and secreted by microglial cells [21,38,39], have the ability to protect neurons and to promote neuronal repair in pathologic-like conditions [11-13]. Although the physiological role of BDNF in neuronal survival/repair has been extensively investigated, the autocrine/paracrine function of BDNF secreted by microglial cells has not been addressed and may actually play a deleterious role. Thus, BDNF can actually behave as a switch by promoting an autocrine action in microglial cells, which may lead to their sustained activation, as occurs in spinal cord cells during neuropathic pain [17]: in this disease, BDNF exacerbates pain hypersensitivity, and strategies to prevent BDNF-mediated microgliosis are considered to have therapeutic potential [18,40]. The present study with cultured N9 microglial cells provides the first direct demonstration for a key autocrine/paracrine role of BDNF in the LPS-induced proliferation of microglial cells: thus, LPS triggers the secretion of BDNF, which we showed to be directly responsible for the proliferation of microglial cells.

The second major novel conclusion of this study was the ability of the A_2A_R blockade to prevent LPS-induced microglial proliferation, as a result of the dual ability of A_2A_R to control the LPS-induced secretion of BDNF and the ability of this BDNF to trigger microglial proliferation. These observations are in agreement with the ability of the A_2A_R blockade to control *in vitro* microglial proliferation [37]; they are also heralded by the numerous reports showing that A_2A_R controls microglial activation, namely responses triggered by LPS [8,41], as well as microgliosis in animal models of inflammatory disease [9,42-44]. In particular, the present results that both the induced secretion of BDNF as well as its impact on microglial cells are dependent on A_2A_R activation, provide the first direct demonstration for a tight interplay between A_2A_R and BDNF signaling in glial cells, extending the previously reported tight interaction between A_2A_R and different neurotrophic factors in the control of neuronal responses [45-47]. In contrast to neuronal interactions between BNDF and A_2A_R, which were revealed in physiological-like processes, the interaction between A_2A_R and BDNF in microglial cells seems to occur selectively under pathologic-like conditions typified by microglial activation. Thus, the effect of A_2A_R is not observed in the absence of LPS and the LPS-induced release of BDNF is notably time-dependent and time-coincident with the increase in A_2A_R density, strongly suggesting a role for the up-regulation of A_2A_R in activated microglia as a trigger for this interaction, in agreement with the previously reported selective role of A_2A_R in the pathophysiology of neuroinflammation [9,44,48-50]. It is important to recognize that the present results are in general agreement with the observation that it is the blockade of A_2A_R that prevents neuroinflammation, which is in clear contrast with the anti-inflammatory effect resulting from A_2A_R activation in the control of peripheral inflammation [6] or central pathological conditions, in particular when peripheral cells invade the central nervous system, as occurs through disruption of the blood–brain barrier, where immunosuppressive actions may be mediated by A_2A_R activation rather than blockade [51,52]. The mechanism underlying this contradiction remains to be elucidated. The impact of A_2A_R in controlling inflammation may depend on the type of immune cell involved, as well as the particular conditions found in the brain parenchyma [8], as previously discussed [53]. Considering that the A_2A_R gene has independent promoters [54], region-specific transcriptional control of A_2A_R may explain differences between central and peripheral immune responses.

**Figure 8:**
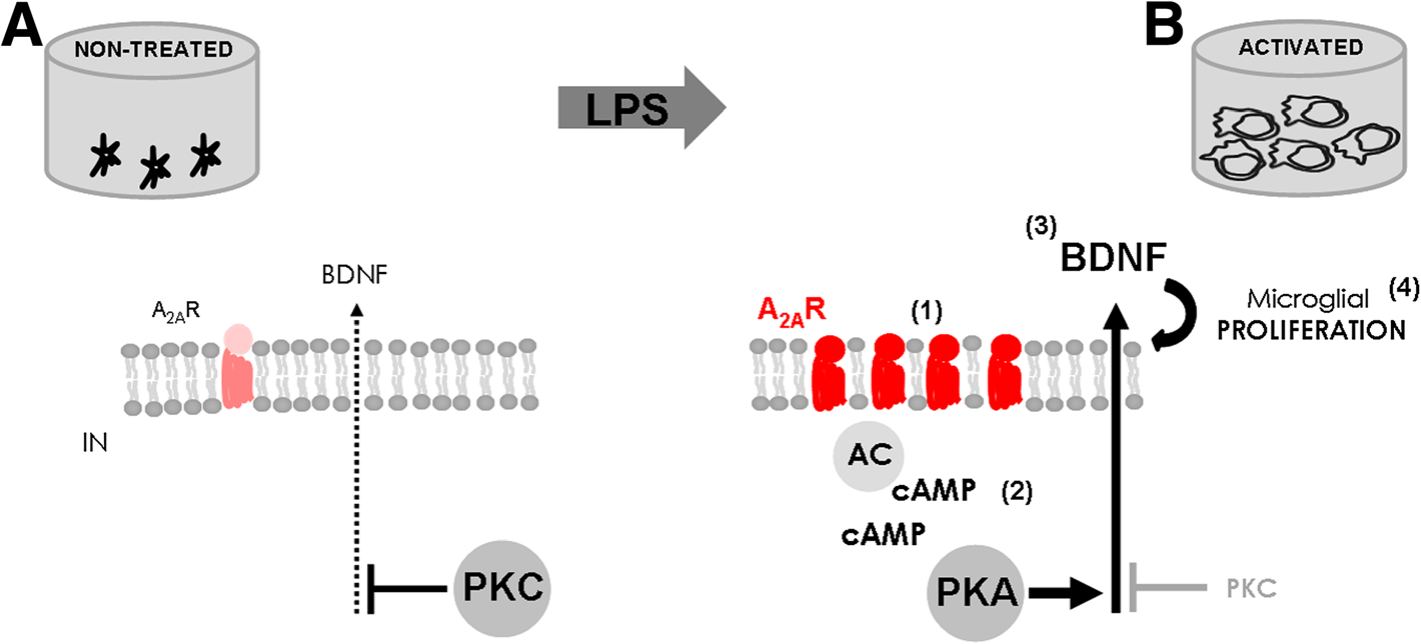
**Schematic representation of the role of A**_**2A**_**R in the control of BDNF release and microglial proliferation selectively in activated microglia. (A)** Constitutive release of BDNF is under the control of PKC (non-treated cells). **(B)** In the presence of an inflammatory trigger, LPS, the increase in A_2A_R density (1) and the subsequent downstream activation of AC-cAMP-PKA (2) prevails over PKC actions, inducing an increase in BDNF release (3), which increases microglial proliferation (4). AC, adenylyl cyclase; cAMP, cyclic AMP; IN, intracellular compartment; PKA, protein kinase A; PKC, protein kinase C.

The final interesting conclusion from this study is the observation that LPS redirects the intracellular pathway involved in the regulation of BDNF secretion from PKC to PKA. Considering the parallel increase in A_2A_R density, this shift is likely due to an increase in A_2A_R signaling. Indeed, the basal outflow of BDNF seems to be affected by a blockade of PKC but not of PKA; in contrast, the LPS-induced outflow of BDNF, shown to be mediated by the endogenous activation of the up-regulated A_2A_R, was prevented by PKA rather than PKC inhibition, suggesting a reorganization of the control of BDNF outflow upon microglial activation by LPS (Figure 8).

## Conclusion

In inflammatory conditions microglial cells are activated and proliferate, which is a beneficial response if self-limited, but can become detrimental if sustained. The present study shows that BDNF secretion upon microglial activation plays an autocrine/paracrine role bolstering microglial proliferation, which may drive sustained microgliosis. Furthermore, we provide the first demonstration that adenosine A_2A_R play a key dual role in the control of BDNF secretion upon microglial activation, as well as on BDNF-induced proliferation of microglial cells. This provides the first direct demonstration for the interplay between A_2A_R and BDNF in glial cells and provides a novel mechanistic insight into the therapeutic potential of A_2A_R antagonists to control neurodegenerative conditions where chronic neuroinflammation is present.

## Abbreviations

A_2A_R: A_2A_ receptor; AC: adenylyl cyclase; ADA: adenosine deaminase; ANOVA: analysis of variance; BCA: bicinchoninic acid; BDNF: brain-derived neurotrophic factor; BrdU: 5-bromo-2'-deoxyuridine; BSA: bovine serum albumin; cAMP: cyclic AMP; ECF: enhanced chemofluorescence reagent; EDTA: ethylenediamine tetraacetic acid; ELISA: enzyme-linked immunosorbent assay; LPS: lipopolysaccharide; mBDNF: mature protein BDNF; PBS: phosphate-buffered saline; PKA: protein kinase A; PKC: protein kinase C; PMSF: phenylmethylsulfonyl fluoride; pro-BDNF: precursor protein BDNF; PVDF: polyvinylidene difluoride; RT: room temperature; SDS: sodium dodecyl sulfate; SEM: standard error of the mean; TBS: Tris-buffered saline .

## Competing interests

The authors declare that they have no competing interests.

## Authors’ contribution

CG was responsible for the quantification of extracellular BDNF (ELISA), intracellular BDNF (Western bloting, with the help of RS), primary cultures of microglia (with the help of JG) and for the experimental design, for coordinating the project and organizing the manuscript with the help and under the supervision of RAC. RF was in charge with proliferation assays and image selection and organization, as well as scientific advice. JG performed Western blot analysis of p38 and IL-1beta detection (ELISA). DR performed Western blot analysis and immunocytochemistry for A2AR. NG performed A2AR signaling experiments. All authors read and approved the final manuscript.
